# The impact of Tai Chi’s “Xuling Dingjin” posture on lumbar biomechanics during stair descent

**DOI:** 10.3389/fspor.2025.1637586

**Published:** 2025-12-05

**Authors:** Yiting Duan, Haibin Liu, Jian Jiang, Liqing Liu, Fan Gao, Suheng Li, Yulong Yang, Shuye Yang, Shudong Yan

**Affiliations:** 1 Spine Surgery Department of the Central Hospital, Dalian University of Technology, Dalian, LiaoNing, China; 2 College of School of Sports and Health Sciences, Dalian University of Technology, Dalian, LiaoNing, China; 3 College of Physical Education and Health, University of Kentucky, Lexington, KY, United States

**Keywords:** Tai Chi, lumbar biomechanics, stair descent, finite element method, opensim

## Abstract

**Background:**

Low back pain is common increases stair-related fall. The Tai Chi “Xuling Dingjin” posture may enhance spinal stability, its biomechanical mechanisms remain unclear. This study investigates the effects of this posture on lumbar biomechanics during stair descent, and provides theoretical support for its application in balance improvement and rehabilitation.

**Research question:**

To investigate the biomechanical effects of Tai Chi's “Xuling Dingjin” posture on the lumbar spine and whether it enhances the stability of the lumbar spine in the staircase environment.

**Methods:**

Twelve adults (6 males and 6 females) with a minimum of 5 years of Tai Chi experience participated in the study. Lumbar biomechanics were assessed during normal stair descent (D) and stair descent incorporating the “Xuling Dingjin” posture (XD) using a Vicon motion capture system, an AMTI force platform, OpenSim biomechanical analysis software, and finite element analysis.

**Results:**

Under the XD condition, deep stabilizing muscles (especially quadratus lumborum) exhibited earlier and more intense activation. Additionally, there was a smaller offset between the center of mass (COM) and center of pressure (COP), indicating improved posture stability. Lumbar rotation around the Z-axis was significantly decreased, and finite element analysis demonstrated a more uniform pressure distribution across the intervertebral discs.

**Conclusion:**

Maintaining the “Xuling Dingjin” posture can activate deep stabilizers earlier and more effectively, redistributing lumbar pressure through postural adjustment, thereby enhancing spinal stability and offering potential value in reducing fall risk.

## 1 Introduction

Stair ascend and descend require coordinated movements of the spine and lower limbs, involving both obstacle navigation and rising from a seated position. Safe stair descent is particularly critical for the elderly and individuals with physical disabilities, as it supports independent living, reduces caregiver burden, and contributes to overall health and quality of life (1). Epidemiological studies indicate falls are prevalent across all age groups, with the risk significantly increasing in older adults—accounting for two-thirds of accidental deaths in individuals over 75 (2, 3). Notably, more than 10% of these falls occur on stairs (4). Stair ascend increases spinal load and motion amplitude, which may exacerbate low back pain (5), while stair descend presents a higher risk of falling due to spinal instability and erratic movements (6). In recent years, there has been growing emphasis on exercise-based rehabilitation for spinal disorder prevention and management (7), with exercise programs promoting spinal health gaining global recognition (8).

Tai Chi, a traditional Chinese martial art, now as a modern competitive sport featuring both routine practice and free sparring, often with an emphasis on Ornamental. Research has shown that Tai Chi integrates the coordination of “mind, breath, and body” (9), enhances perceptual awareness through cognitive engagement (10), stabilizes the spine via abdominal breathing (11), and improves posture by promoting vertical spinal alignment (12). It has also been demonstrated to enhance dynamic balance and reduce fall risk (13), making it effective in improving physical fitness and preventing diseases.

“Xuling Dingjin” is central to Tai Chi form and body alignment. “Xuling” denotes a relaxed and supple state of the head and neck; “ding” refers to a gentle, intentional upward lift of the crown, as if a force were drawing the baihui acupoint toward the sky (14). “Jin” arises from a spiraling interplay of muscle, bone, and connective tissues—alternating stretch and compression—that converts mechanical energy into elastic potential (15). During “Xuling Dingjin” practice, this elasticity is not confined to the limbs; it flattens the physiological spinal curves, shifting the spine from an “S”-shape toward a “C”-shape and ultimately toward a straight axis. This reconfiguration is expected to distribute disc loads more evenly and to provide effective conditioning of the deep spinal musculature (16). “Xuling Dingjin” integrates core concepts such as “containing the chest and pulling out the back”, “standing upright in the middle” and “Qichen Dantian” (engaging core stability) (17). Biomechanically, this posture transforms the spine into a flexible chain, with the cervical vertebrae acting as a fixed anchor and the lumbar vertebrae free to elongate and align. This configuration is thought to lengthen the spine and improve segmental aligning. However, the physical effects of maintaining the “Xuling Dingjin” posture are often described subjectively, and there is limited empirical data to support its biomechanical benefits.

To address this gap, previous studies have explored Tai Chi from various perspectives. For example, Law et al. (18) explored the muscle activation characteristics of seven types of Tai Chi forms, Hass CJ et al. (19) examined how the center of pressure (COP) contributes to the center of mass (COM) stability, and Zhao L et al. (20) developed an L4–L5 spine model to investigate the biomechanical effects of the “cloud hand” maneuver. Building on this foundation, the present study is the first to quantify biomechanical characteristics of the lumbar spine during the “Xuling Dingjin” posture. Our study aims to determine whether this posture could make stair descent safer and biomechanically less demanding. The hypotheses of this study are: (1) “Xuling Dingjin” posture can enhance the activation of the paraspinal muscles, thereby improving lumbar stability; and (2) maintaining the “Xuling Dingjin” posture during stair descent can reduce the angle of lumbar curvature, thereby provides a safer way of traveling.

## 2 Methods

### 2.1 Participants

Twelve healthy adults were recruited for this study (6 males, age 41.3 ± 8.8, height 173.7 ± 5.16 cm, mass 74.71 ± 8.2 kg; 6 females, age 54.3 ± 3.2, height 162.2 ± 2.8 cm, mass 58.3 ± 8.2 kg). Inclusion criteria were: healthy, ≥5 years of continuous Tai Chi practice, no recent surgery or illness, and the ability to perform Tai Chi movements accurately. All participants were familiar with the “Xuling Dingjin” posture. The researchers recorded the age, gender, and years of tai chi training, and measured the height, weight, leg length, arm length, shoulder width, elbow width, wrist width, and ankle width data of all participants. All participants signed an informed consent form prior to the trial, and it was approved by the ethics committee.

### 2.2 Procedures

**Figure 1:**
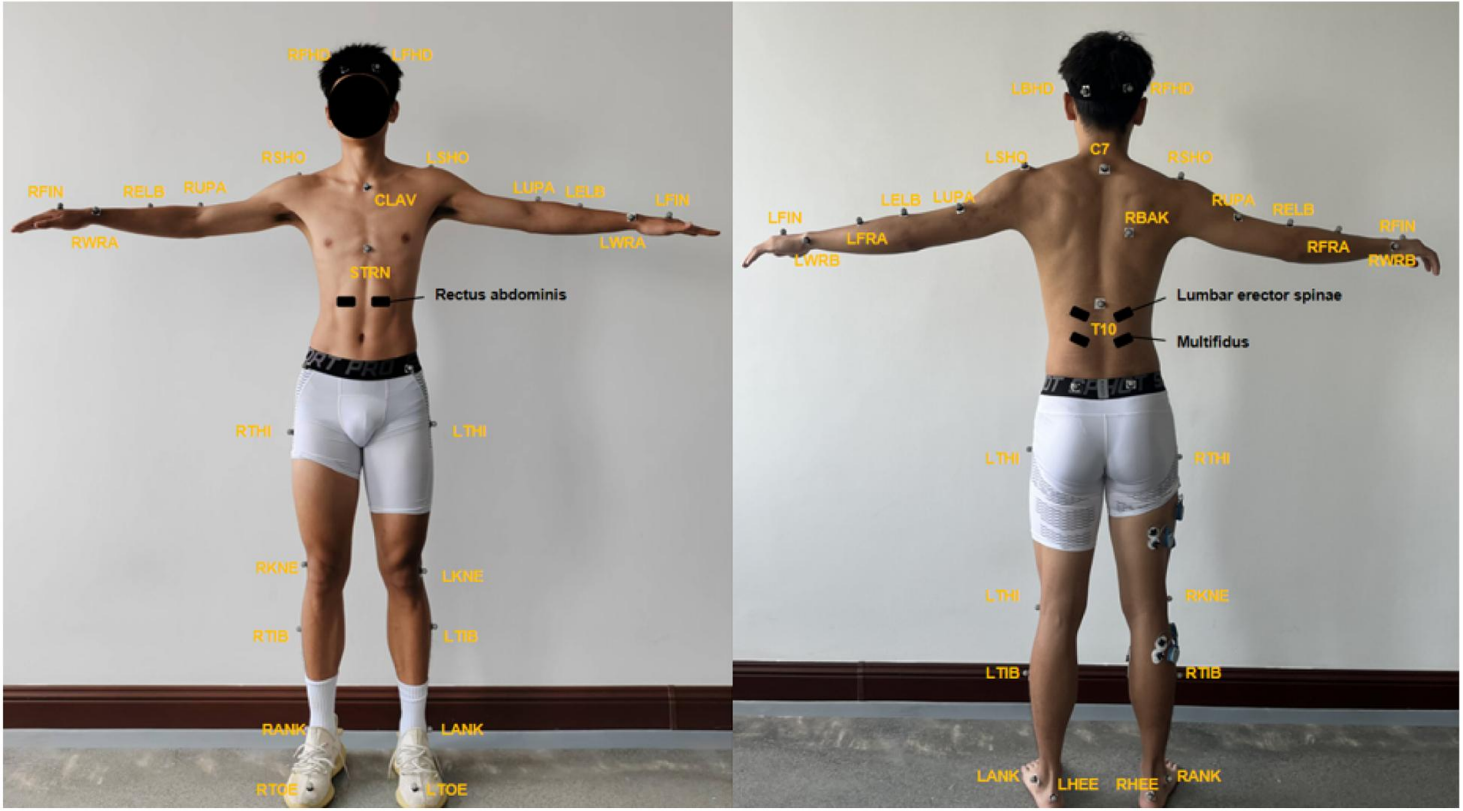
39 markers in body joint points.

The experimental setup featured a custom-built two-step staircase with a step height of 17 cm, consistent with dimensions for residential building stairs. Each step was equipped with an AMTI force platform (Optima HPS, AMTI, 103 USA), sampling at a frequency of 1,000 Hz to collect kinetic data. Kinematic data were collected using a Vicon motion capture system (Vicon 99 V5, Oxford Metrics, UK) with eight infrared cameras operating at 100 Hz, that was synchronized with the force plates. Thirty-nine reflective markers were placed on bony landmarks of the head, trunk, pelvis, bilateral upper limbs, bilateral lower limbs, and feet, following the Vicon Full-body AI model. Electromyographic data from the multifidus and psoas major muscles were recorded using a Noraxon Surface Electromyography (sEMG) system at 1,500 Hz (Figure 1).

Participants wore standardized tight-fitting clothing and Tai Chi shoes. To ensure consistent gait and speed, all participants started from the top step and descended using their right (the traditional kickball method was used to determine that the dominant sides of the subjects were all right-sided) foot in each trial, guided by a metronome (0.5 m/s). The study employed a blinded design: subjects first performed five trials of normal stair descent (D), followed by a 5 min rest. They were then instructed to complete five additional trials while maintaining the Tai Chi “Xuling Dingjin” posture (XD).

### 2.3 Data acquisition and analysis

Muscles activation data for paravertebral muscles were derived using Opensim software (version 4.4, Stanford University, USA) through static optimization and time normalization. To validate OpenSim outputs, raw sEMG signals were processed using a Butterworth bandpass filter (10–500 Hz) and 50 Hz notch filter. The signals were then rectified, normalized, and RMS quantized for the multifidus muscle, and smoothed using a 50 ms sliding window. These processed sEMG activation curves were compared with Opensim-simulated muscle activations (21).

The trajectories of the COP were computed from the ground reaction force, specifically from the moment the right foot left the top step until the left foot contacted the lower step. The excursion of COP trajectory in the sagittal (X) and coronal (Y) planes was calculated as the maximal displacement in each direction. COM was calculated by Vicon software from anthropometric data. Both COM and COP were normalized across participants to take into account interindividual variability. Posture stability was assessed by the offset between COM and COP trajectories (22, 23).

Lumbar spine L4–5 angles in flexion-extension (X), lateral bending (Y), and axial rotation (Z) were derived using inverse kinematics and normalized for comparison (24).

**Figure 2:**
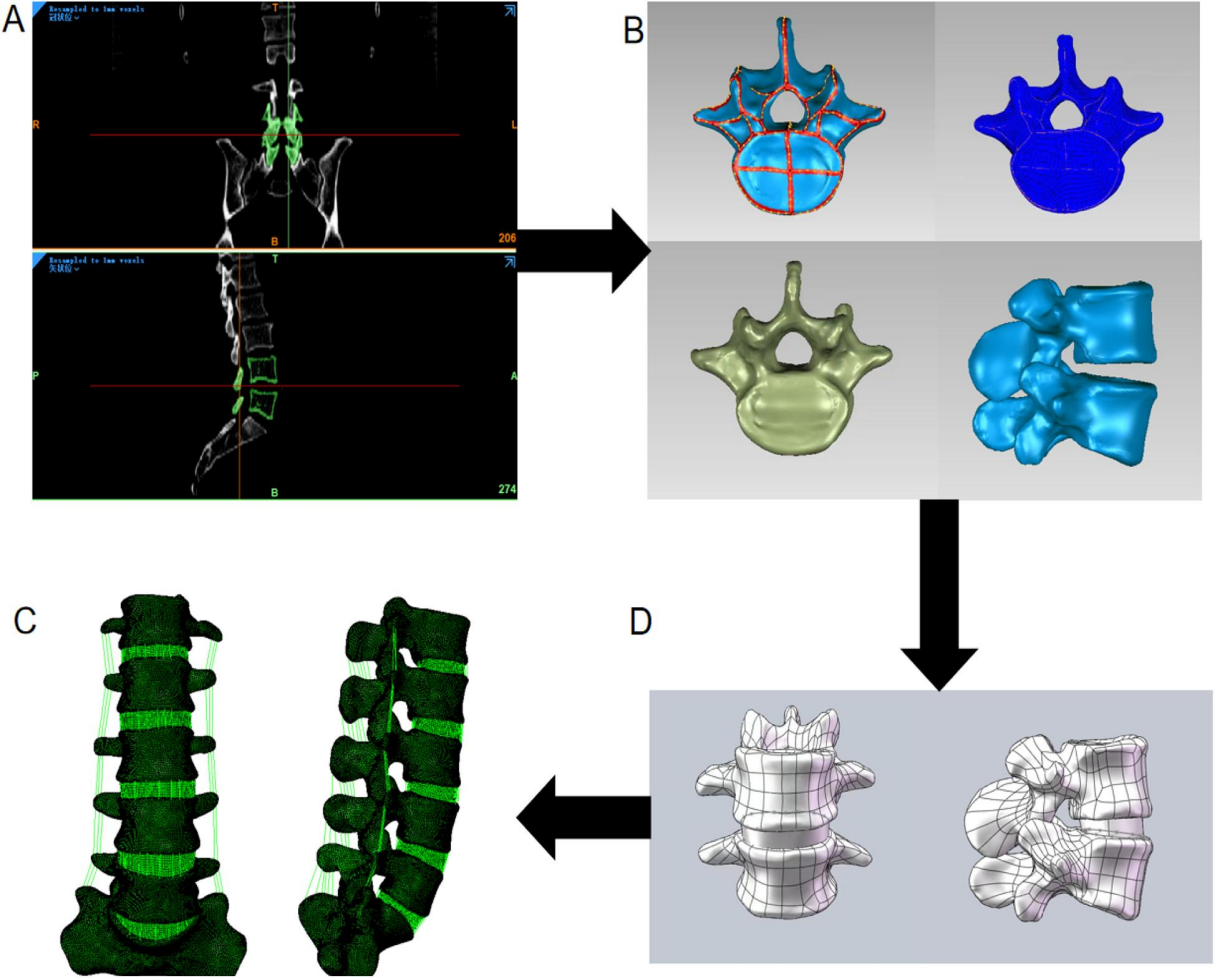
Schematic diagram illustrating the construction process of the finite element model of the L1–5 lumbar spine. **(A)** Bony structures were segmented and reconstructed using Mimics software; **(B)** Surface smoothing and noise removal were performed using Geomagic Wrap software; **(C)** Intervertebral discs were modeled and assembled with vertebral structures in Solidworks software; **(D)** Ligaments were added, the model was meshed, and finite element analysis was conducted in ABAQUS software.

For finite element analysis, the lumbar spine (L1–L5) of the participant with the most advanced Tai Chi proficiency was scanned using CT, and DICOM images were imported into Mimics 21.0. Vertebrae were segmented via masking and thresholding, gaps filled, and the model exported as an STL file. Geomagic Wrap 2021 was used to smooth the model (grid doctor, spike removal, hole-filling), separate cancellous and cortical bone (2 mm cortical reference), and saved the result in STP format. Intervertebral disc (including end-plate cartilage, annulus fibrosus, nucleus pulposus) were modeled in SolidWorks using surface offset and Boolean operations, and performed hexahedral mesh generation. The complete SLDPRT model was analyzed in Abaqus Finite Element Analysis | SIMULIA, with appropriate material properties and apply a fixed constraint to the base surface of the lumbar vertebra L5 segment, restricting all degrees of freedom. OpenSim calculated lumbar reaction force and torque corresponding to the GRF peak moment was applied to simulate stress distribution in the intervertebral discs. The finite element modelling process is shown in Figure 2, and the material properties of all parts are taken from references (25, 26) and listed in Table 1.

**Table 1:** Paravertebral muscle activation peaks and troughs.

Tissue	Element type	Young's modulus (MPa)	Poisson's ratio
Cortical bone	Tetrahedron	12,000	0.3
Cancellous bone	Tetrahedron	100	0.2
Cartilage	Tetrahedron	35	0.4
Marrow	Hexahedron	4	0.45
Annulus fibrosus matrix	Hexahedron	4.5	0.4
Annulus fibrosus	Hexahedron	550	0.3
Anterior longitudinal ligament	Spring	7.8	–
Posterior longitudinal ligament	Spring	10	–
Interspinous ligament	Spring	10	–
Supraspinous ligament	Spring	8	–
Lateral ligament	Spring	10	–
Ligamentum flavum	Spring	10	–
Muscle	Spring	–	–

All data were analyzed for normality using GraphPad Prism 9.5.0. Paired t-tests were used for normally distributed data, while Wilcoxon signed-rank tests were used for non—normal data. *Indicates *P* < 0.05, ** indicates *P* < 0.01. All data were normalized using Origin.

## 3 Result

### 3.1 Muscle activation validation

**Figure 3:**
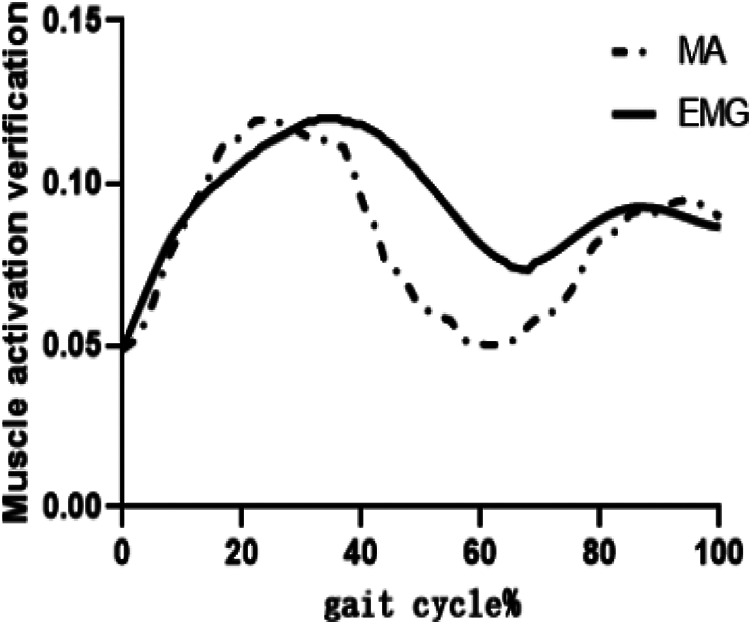
Validity verification by comparing between muscle electrical stimulation results and opensim muscle activation simulation results.

Paravertebral muscles activation are derived using Opensim software (version 4.4, Stanford University, USA) after static optimization followed by time normalization. To verify the reliability of the results, the measured sEMG signals were filtered by Butterworth bandpass filter (10–500 Hz) and 50 Hz notch filter, rectified, normalized, and RMS quantized for the multifidus muscle. Finally, the data were smoothed using a 50 ms sliding window to generate the activation curves, which were compared with the muscle activation curves simulated by Opensim (21, 27). The muscle activation predicted by the Opensim model and the experimental EMG average graph (Figure 3) show that the overall trends of the muscle activation simulated by Opensim and the processed EMG are approximately the same, indicating that the simulation results are reliable.

### 3.2 Muscle activation

**Figure 4:**
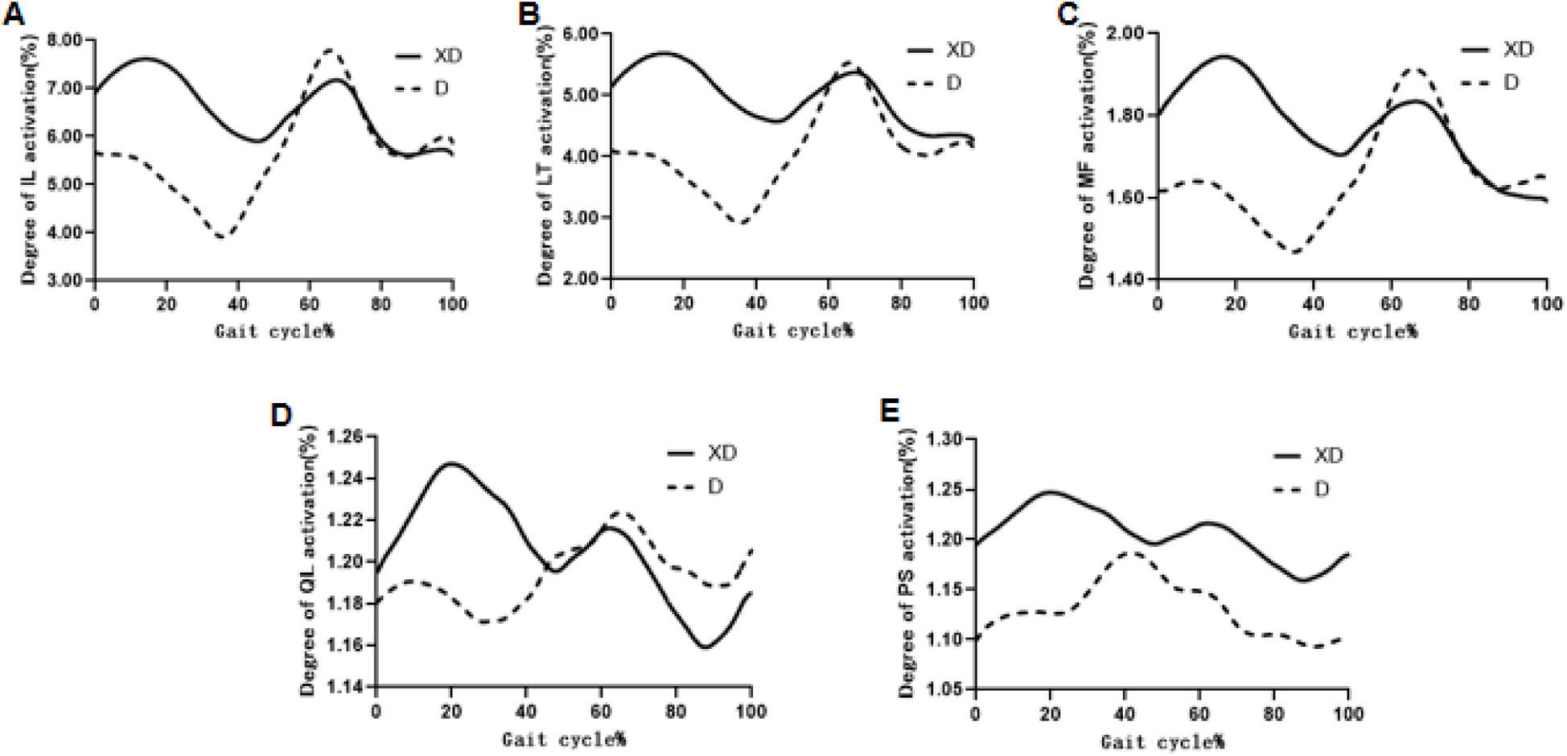
Comparison of muscle activation of the paraspinal muscles of the stair descent in both conditions. **(A)** IL, **(B)** LT, **(C)** MF, **(D)** QL, **(E)** PS.

**Figure 5:**
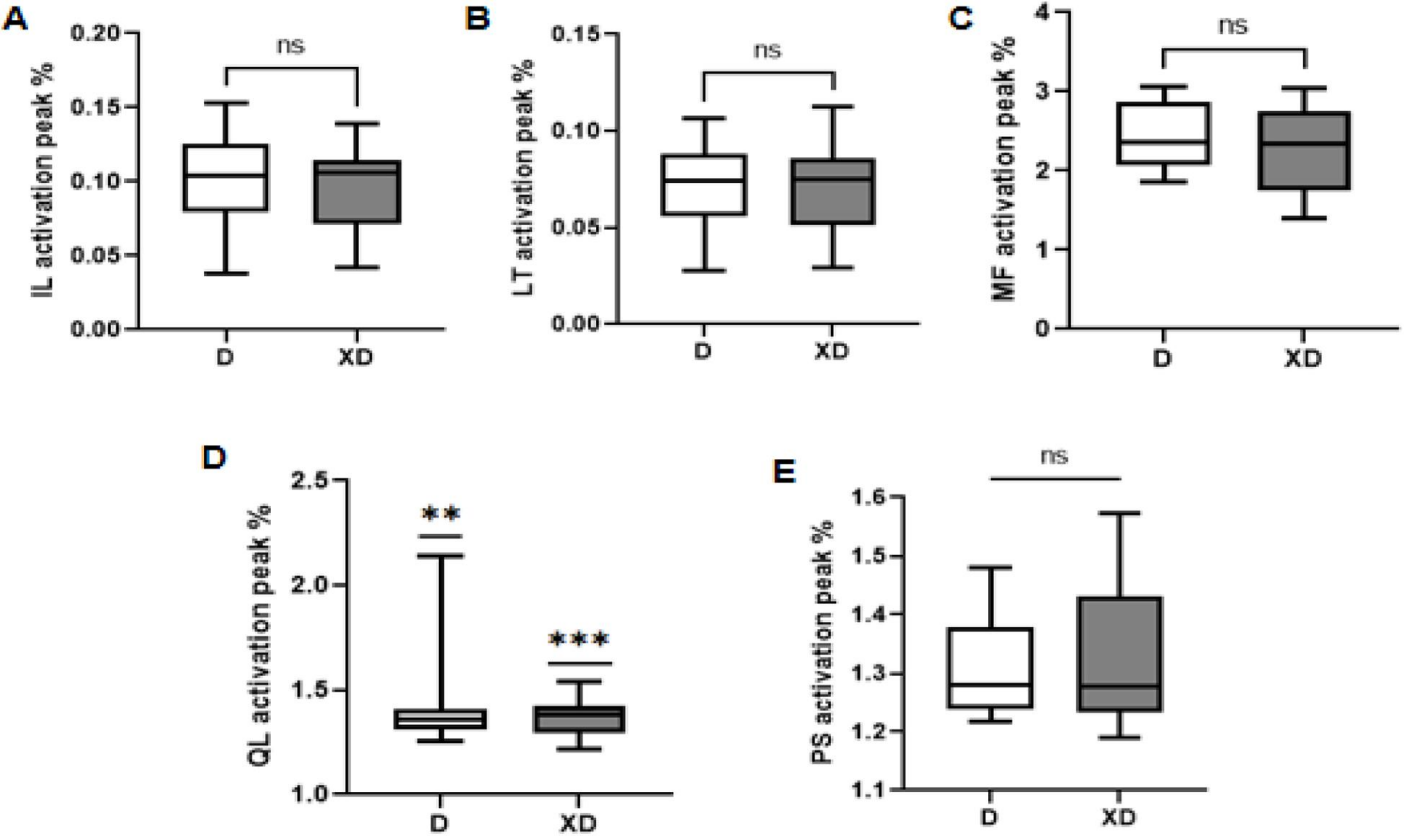
Comparison of peak muscle activation of paravertebral muscles in D and XD. **(A)** IL, **(B)** LT, **(C)** MF, **(D)** QL, **(E)** PS.

During both D and XD, the iliococcygeus (IL), longissimus (LT), multifidus (MF), and quadratus lumborum (QL) showed a biphasic activation pattern. In contrast, the psoas major (PS) showed a single activation plateau during D (at 40%–60% of the gait cycle). For IL, LT, MF, the maximum activation during D occurred at the second peak (IL: 10.128 ± 3.087, LT: 7.258 ± 2.306, MF:2.424 ± 0.405) whereas during XD it shifted to the first peak (IL:9.751 ± 2.65, LT:7.468 ± 0.246, MF: 2.287 ± 0.499). PS activation was consistently higher during XD (peak angle:1.330 ± 0.121) than D (peak angle:1.310 ± 0.083). In addition, QL had significantly greater muscle activation in XD (1.327 ± 0.085) compared to D (1.468 ± 0.246) (*P* = 0.002), while no significant differences were observed in other muscles (*P* > 0.05) (Figures 4, 5).

### 3.3 Relative position analysis of COP and COM

**Figure 6:**
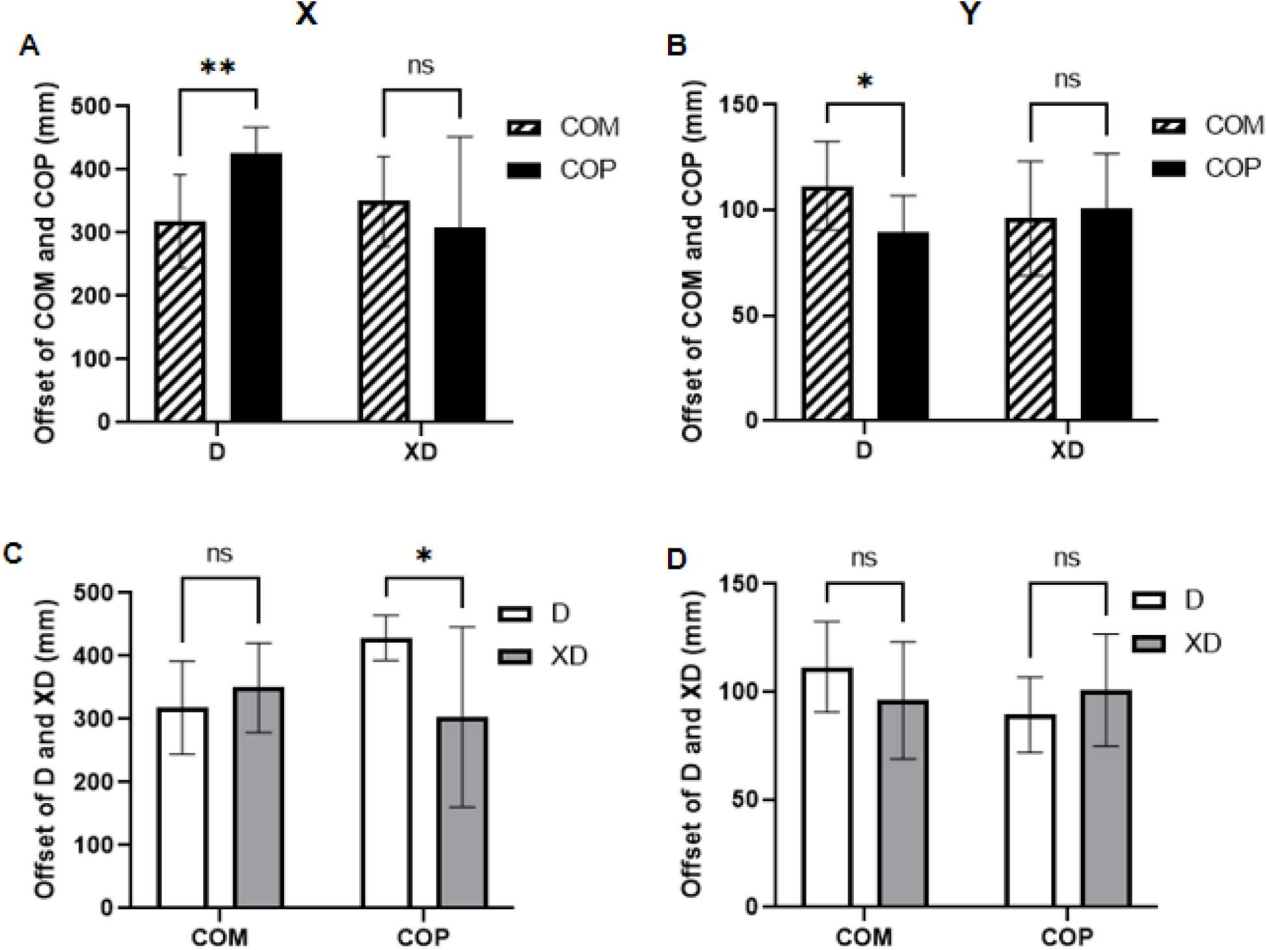
Comparison of COP and COM displacement during stair descent between D and XD. **(A)** Comparison of COP and COM maximum offset along *X*-axis; **(B)** Comparison of COP and COM maximum offset along *Y*-axis; **(C)** COM and COP offset between D and XD along *X*-axis; **(D)** COM and COP offset between D and XD along *Y*-axis.

The two stair descent conditions affected the relationship between the center of mass (COM) and center of pressure (COP) differently. In D, there were significant differences between COM and COP displacements in both the sagittal (X) and coronal (Y) planes, indicating less stability. In contrast, no significant differences were observed in XD. In the X direction, COP displacement was significantly larger in D than XD, while COM displacement was smaller but not significantly different. In the Y direction, no significant differences in either COM or COP were found between D and XD (Figure 6).

### 3.4 Lumbar spine kinematics

**Figure 7:**
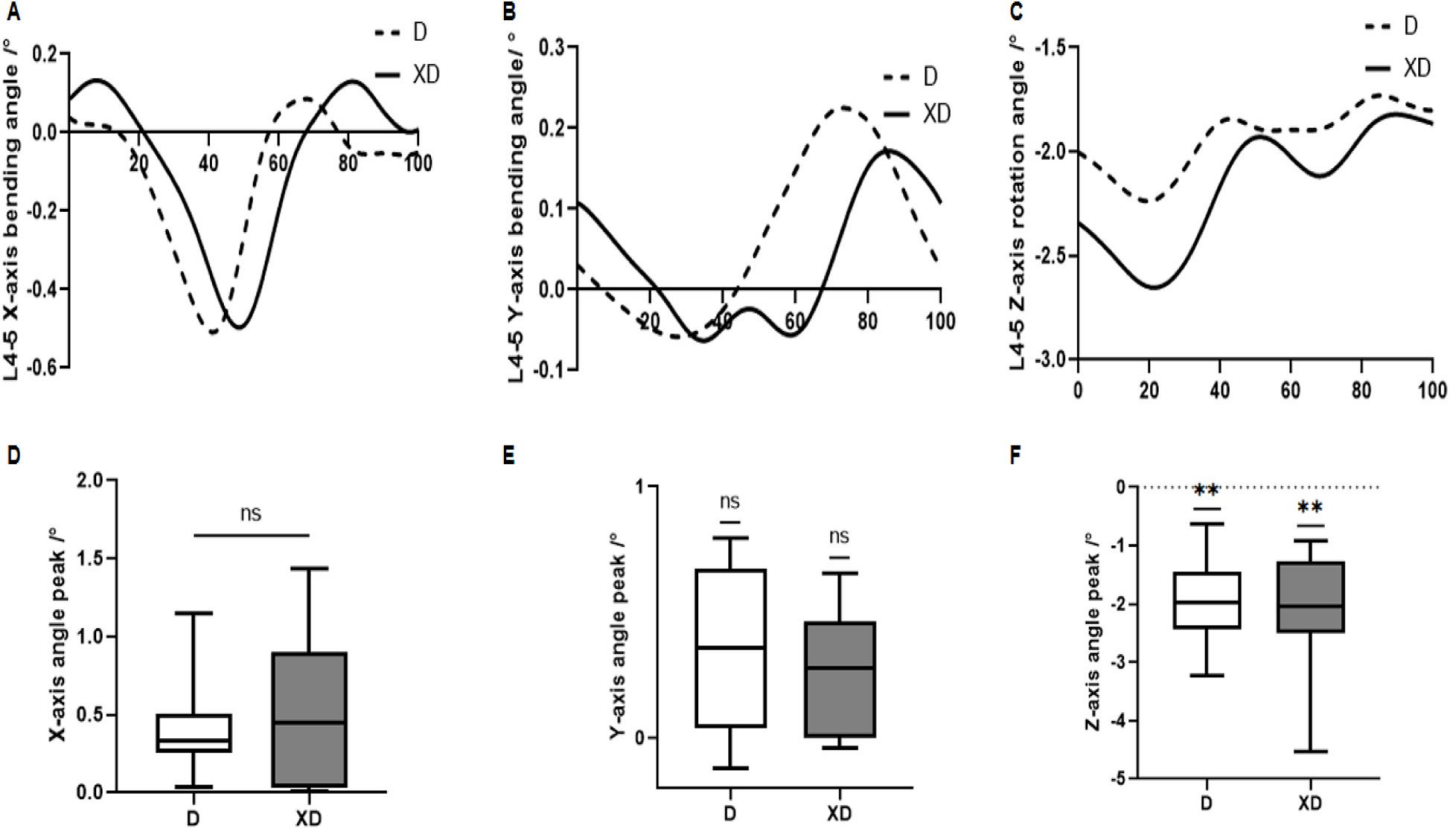
L4–5 motion comparison during stair descent between D and XD. **(A)** Flexion/extension (*X*-axis), **(B)** Lateral bending (*Y*-axis), **(C)** Axial rotation (Z-axis), **(D)** Peak *X*-axis angle, **(E)** Peak *Y*-axis angle, **(F)** Peak Z-axis angle.

The movement patterns of the L4–5 in the X, Y and Z directions were similar across both D and XD conditions. No significant differences were found in *X* axis (D: −0.419du ± 0.308, XD: −0.488 ± 0.477) or *Y* axis (D:0.387° ± 0.272°, XD: 0.281° ± 0.208°). However, the rotation angle around the Z-axis was significantly reduced in XD (−2.096 ± 1.025) compared to D (−1.957 ± 0.682) (*P* = 0.002) (Figure 7).

### 3.5 Model validation

**Figure 8:**
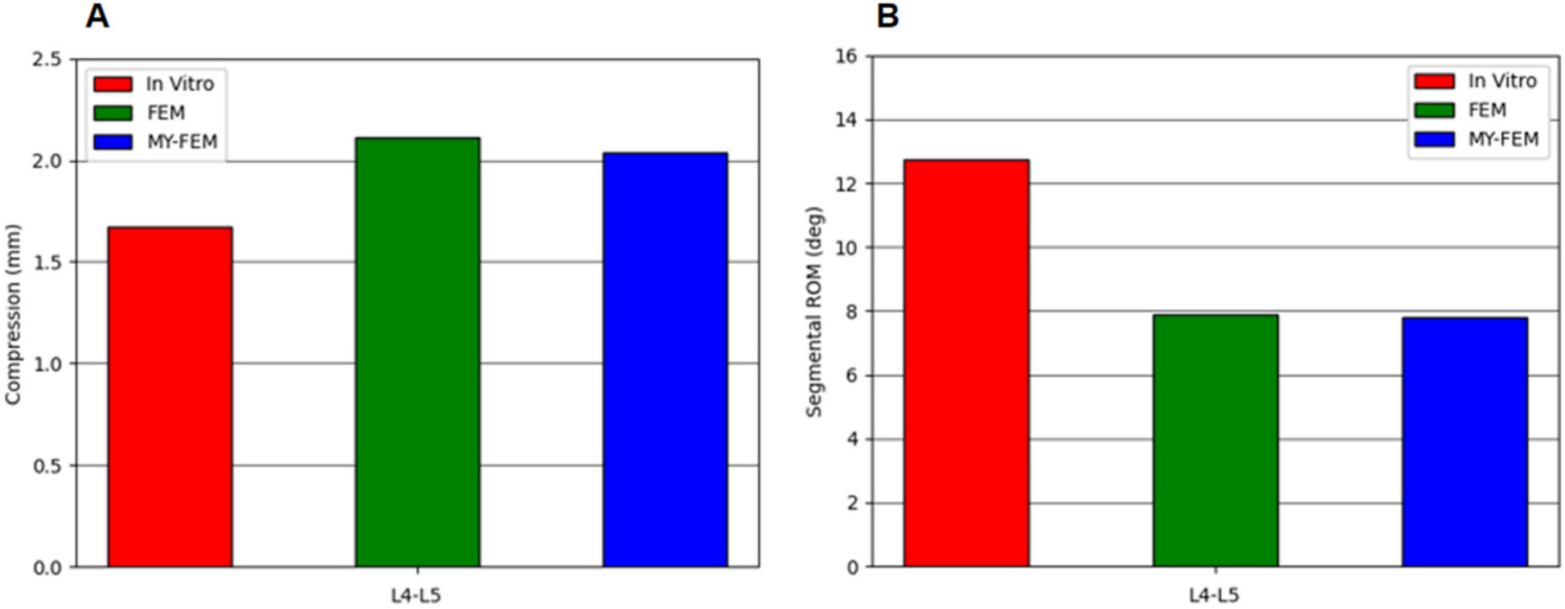
Finite element model verification results. **(A)** Lumbar compression displacement, **(B)** Lumbar flexion-extension displacement.

To validate our model, we applied loading parameters and boundary conditions from Renner S. M et al. (28), in their study, lower end of the fifth lumbar vertebra of the fixed cadaver specimen was preloaded with a vertical load of 1,200 N and a flexion-extension torque of 8 Nm at the top of the first lumbar vertebra, and the results were compared with the simulation data from the established finite element model. The same loads and boundary conditions were applied to the finite element model in this study, and the simulation data were compared with the reference literature data. The results are shown in Figure 8, where the model results are similar to those in the literature, indicating that the model is valid.

### 3.6 Finite element analysis of lumbar intervertebral discs

**Figure 9:**
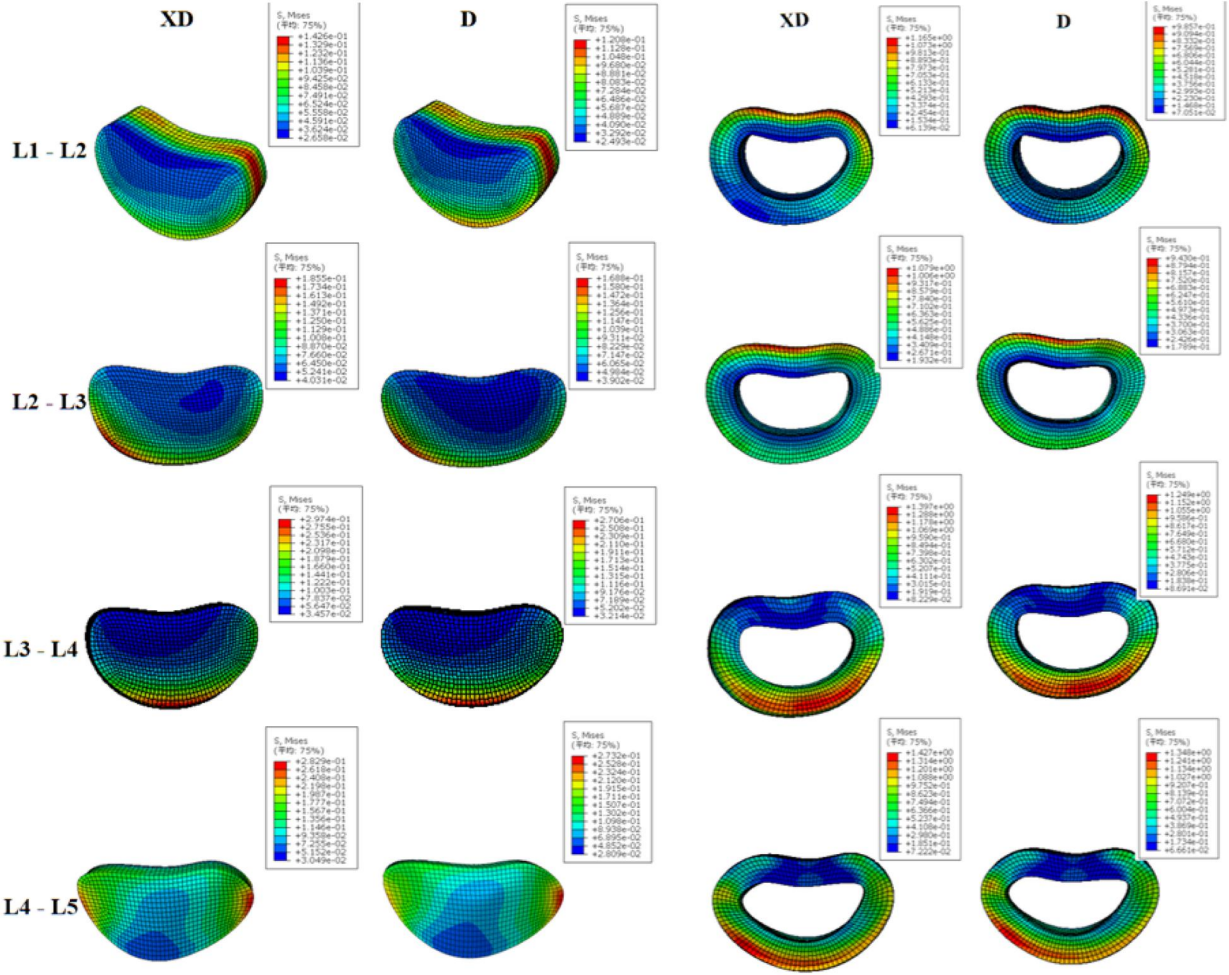
Stress distribution of the L1–5 medulla and the annulus fibrosus for both stair-descent approaches.

Static optimization and finite element analysis were used to assess the stress distribution across the lumbar intervertebral discs during stair descent. Peak ground reaction forces and torques form both D and XD contions were applied to the lumbar spine model. In the nucleus pulposus, XD produced higher stress than D. For L1–2, stress in XD concentrated posteriorly, whereas in D it appeared at both the anterior and posterior regions. In L4–5 Stress, D showed unilateral stress (left side), while XD exhibited a more symmetrical distribution. The maximum stress area was larger in D than in XD. In the annulus fibrosus, although the peak XD pressure is generally 0.01 MPa higher, the overall stress area is larger compared to D, which has a smaller maximum stress distribution (Figure 9).

## 4 Discussion

Previous studies examining the effects of Tai Chi on the spine have been largely limited to textual descriptions and lacked theoretical basis. In this study, we focused on exploring the core principle of Tai Chi, the state of “Xuling Dingjin”, and quantified its biomechanical characteristics. Our findings demonstrated that this posture can help stabilize the lumbar spine during stair descend, potentially reducing the risk of injury.

In the natural stair descent condition (D), paraspinal muscles exhibit a bimodal activation pattern, with peaks at approximately 25% (right leg swing phase) and 60%–80% (right leg stance, left leg swing) of the gait cycle, and a trough at around 40% (right foot contact, left leg initiation). This pattern aligns with human motion datasets by Camargo et al. (Human Motion Dataset) (29) and reflects varying mechanical demands. The second peak, in particular, supports lumbar stability as the center of gravity shifts backward and the lower limbs extend, with eccentric contractions aiding in impact absorption and trunk balance, as noted by Muscle Contraction Study (30).

In contrast, during the Tai Chi “Xuling Dingjin” (XD) condition, peak muscle activation shifts to 20%–40% of the gait cycle, indicating earlier recruitment of deep stabilizers like multifidus (MF) and quadratus lumborum (QL) during initial foot contact. This aligns with Tai Chi's principle of “using intention, not force,” which emphasizes neuromuscular coordination and movement efficiency (18). While the psoas (PS) muscle also showed greater activation in XD (though not statistically significant), it plays a vital role in posture control, spinal stabilization, and hip flexion during contralateral weight-bearing, consistent with findings from Hodges (Psoas Function Study) (31) and Tai Chi's “XuLing DingJin” emphasis on spinal elongation. Notably, QL exhibited significantly greater activation in XD (*P* < 0.01), supporting its functions in spinal extension, lateral bending, and rotation. The enhanced activation contributes to better core stability and balance control in the lower limbs, key components of the “Xuling Dingjin” principle. Collectively, these findings suggest that the XD posture improves spinal stability during functional tasks, and may potentially reduce injury risk.

Asynchronous trunk flexion and lower limb propulsion in the D group during stair descent caused significant displacement in both X and Y directions, (Figures 5A,B), altering lumbar torque and increasing the COM-COP phase difference (32, 33). In contrast, the XD group showed no significant differences, likely due to enhanced spinal stability associated with the XD posture. Furthermore, the D group had a significantly larger COP displacement than the XD group (Figure 5C), indicating increased forward foot pressure without proportional trunk flexion. This reflects a compensatory “braking strategy” commonly observed in older adults (34), where heel pressure is shifted backward to counterbalance trunk lean and prevent falls. The spatial relationship between COM-COP is crucial for postural control (35). Improved alignment and reduced displacement under SD suggests enhanced neuromuscular coordination and dynamic balance, hallmarks of Tai Chi practice (36).

Comparing the two stair descent conditions, no significant differences were found in lumbar flexion/extension or lateral bending angles. However, the XD posture significantly reduced L4–L5 rotation toward the left, indicating that the trunk shifted toward the right—that is, toward the advancing right leg—thereby decreasing the angle between the spine and the leg. Muscle activation patterns further revealed earlier peak activation (20%–40% gait cycle) in the iliocostalis (IL), longissimus thoracis (LT), multifidus (MF), and quadratus lumborum (QL) under XD, with QL showing significantly higher activation than in D. These findings align with the Tai Chi practice “Qichen Dantian”, which emphasizes trunk stability through core engagement. Previous studies show that Tai Chi improves lower-limb stability in senior women, reducing fall risk (37). Excessive lumbar rotation is linked to chronic low back pain and disc injury (38), and impaired equilibrium (39). The XD posture may mitigate these risks by restricting lumbar rotation and optimizing muscle activation.

Finite element model showed that the intradiscal pressure in the D condition was about 0.1 MPa higher than in the XD condition, a minor difference relative to typical intradiscal pressures (0.5 MPa standing, 1.0–2.3 MPa during activities) (40). However, pressure distribution was more uniform in the XD condition, which had a larger *X*-axis angle and smaller Z-axis range of motion at L4–L5, suggesting reduced localized stress. In contrast, the D condition exhibited a smaller pressure-concentrated area, which may relate to the observed lumbar motion pattern.

Uniform pressure distribution is more critical than peak pressure in preventing disc injury (41). Localized pressure peaks are associated with chronic low back pain and functional impairments (42). These biomechanical findings are consistent with Tai Chi principles such as ‘Xuling Dingjin’ and ‘Songyao Luokuang’, emphasizing natural spinal alignment and even force distribution to reduce stress concentrations. Regarding older adults, Muscle function degradation and pain interference in older adults may lead to lower activation peaks or delayed timing (43). The “Xuling Dingjin” posture not only activates the back muscles but also induces a pre-activation state in the muscles, serving as a potential intervention measure. This posture can serve as a low-intensity, non-invasive intervention in daily training, integrated into balance training programs. By instructing practitioners to maintain the “Xuling Dingjin” posture during stair descent, core stability and neuromuscular coordination can be enhanced, thereby reducing the risk of falls associated with lumbar instability. For patients with lower limb or lumbar issues, such as chronic low back pain or balance disorders, this posture can be incorporated into functional rehabilitation training to optimize spinal alignment and muscle activation patterns, potentially alleviating pain and improving dynamic balance. In summary, we recommend incorporating Tai Chi exercises, particularly the “Xuling Dingjin” movement, into clinical practice in daily life, enabling patients to consciously maintain it during walking or stair navigation.

## 5 Study limitations

This study's small sample size limits the generalizability of the findings. Future research should involve larger cohorts for more robust statistical analysis. Additionally, the finite element model assumed uniform intervertebral disc material properties and fixed boundary conditions, overlooking individual anatomical variations and/or dynamic loading complexities. To addres this, future work will include multiple lumbar models representing different morphologies and conditions. In this study, finite-element modelling of spinal stress employed static loading, however, stair descent is a dynamic task. Future work will therefore incorporate dynamic simulations to more accurately characterise the underlying movement mechanisms. The stair descent task was selected to emphasize lumbar motion and loading, allowing clearer biomechanical differentiation between conditions. Our results suggest the “Xuling Dingjin” posture may improve stair descent safety. Future research will explore its influence across a broader range of movements. These insights offer valuable implications for clinical rehabilitation and Tai Chi-based training.

## 6 Conclusion

Compared with natural stair descent, maintaining the “Xuling Dingjin” posture significantly activates deep stabilizing muscles earlier, promotes lumbar pressure distribution, thereby enhancing spinal stability and offering potential value in reducing fall risk. These findings offer a theoretical basis for incorporating Tai Chi movements into balance training, rehabilitation programs and movement instruction. Future research should further explore the effectiveness of “Xuling Dingjin” in improving dynamic stability and preventing falls, particularly in the elderly.

## Data Availability

The raw data supporting the conclusions of this article will be made available by the authors, without undue reservation.
